# Dietary self-control influences top–down guidance of attention to food cues

**DOI:** 10.3389/fpsyg.2015.00427

**Published:** 2015-04-13

**Authors:** Suzanne Higgs, Dirk Dolmans, Glyn W. Humphreys, Femke Rutters

**Affiliations:** ^1^School of Psychology, University of BirminghamBirmingham, UK; ^2^Department of Experimental Psychology, University of OxfordOxford, UK; ^3^Department of Epidemiology and Biostatistics, VU University Medical CenterAmsterdam, Netherlands

**Keywords:** attention, working memory, food cues, successful self-control, restraint, disinhibition

## Abstract

Motivational objects attract attention due to their rewarding properties, but less is known about the role that top–down cognitive processes play in the attention paid to motivationally relevant objects and how this is affected by relevant behavioral traits. Here we assess how thinking about food affects attentional guidance to food items and how this is modulated by traits relating to dietary self-control. Participants completed two tasks in which they were presented with an initial cue (food or non-food) to either hold in working memory (memory task) or to merely attend to (priming task). Holding food items in working memory strongly affected attention when the memorized cue re-appeared in the search display. Tendency towards disinhibited eating was associated with greater attention to food versus non-food pictures in both the priming and working memory tasks, consistent with greater attention to food cues *per se*. Successful dieters, defined as those high in dietary restraint and low in tendency to disinhibition, showed reduced attention to food when holding food-related information in working memory. These data suggest a strong top–down effect of thinking about food on attention to food items and indicate that the suppression of food items in working memory could be a marker of dieting success.

## Introduction

Motivational objects, such as food cues, can have a strong influence on attention. For example, food items can pop out from visual arrays in search tasks, especially when one is hungry (Mogg et al., 1998). This ability of food to attract attention may be linked to its rewarding properties, which act to ‘drive’ attention to food in a bottom–up manner; food items attract attention because they are perceptually salient (Robinson and Berridge, 1993; Castellanos et al., 2009). However, recent evidence from our laboratory suggests that higher level cognitive processes also direct attention to food, such that merely thinking about food can modulate the extent to which it captures attention; “top–down” modulation of attention (Higgs et al., 2012; Rutters et al., 2014). The proposed mechanism underlying this effect is that holding specific information in working memory causes attention to be involuntarily drawn to similar stimuli in subsequent search displays (Soto et al., 2005; Soto and Humphreys, 2007). These results suggest that attentional biases towards food cues are mediated, at least partly, through working memory as well as acting through bottom–up attentional capture (e.g., the mere priming of the identification system by seeing the food cue).

Some people respond more strongly to food cues than others. People who reduce energy intake to lose weight (dieters) and those prone to overeating show enhanced attention to food-related cues (Green and Rogers, 1993; Brignell et al., 2009). Why this is the case is poorly understood and to date there has been no examination of whether trait motivations affect attentional guidance from working memory, since only non-dieting participants were recruited in previous studies. This is important because (1) it extends our understanding of the factors that affect working memory in guiding attention and (2) attentional bias towards food cues may predict overeating, which is risk factor for future weight gain (Yokum et al., 2011). In the current study, we examined how attention to food items is modulated by individual variation in successful dietary self-control.

There is evidence to suggest that dieting status moderates bottom–up attentional bias to food cues: dieters often show greater bias towards food than non-dieters. Findings are, however, not always consistent perhaps because the tasks used tap into different underlying cognitive functions, which may modulate the effects of food cues on cognition (Green and Rogers, 1993; Hollitt et al., 2010; Meule et al., 2012). Dieting status also moderates the content of working memory, which in turn guides attentional selection. Dieters are reported to have preoccupying thoughts about food (Jones and Rogers, 2003) and these thoughts are known to draw on working memory resources (Kemps and Tiggemann, 2005). However, dieters differ in the extent to which they are successful in controlling their intake and this is related to the traits of disinhibition (the tendency to overeat in the presence of tempting food cues) and restraint (the ability to exert cognitive control over eating) that can be assessed by the Three Factor Eating Questionnaire (TFEQ; Stunkard and Messick, 1985). Participants with high restraint and high disinhibition scores are often referred to as ‘unsuccessful dieters,’ while those with high restraint and low disinhibition scores are often referred to as ‘successful dieters’ (Yeomans et al., 2004). We predict that unsuccessful dieters might show both stronger bottom–up priming, if food representations are strongly pre-activated by the sight of food in dieters, as well as showing greater top–down attentional guidance to food-related stimuli, since unsuccessful dieters may already be thinking about food. In contrast, successful dieters might be less responsive to food cues because they are able to suppress food items in working memory. If this were the case then the results would identify a novel mechanism underlying successful dietary self-control.

To examine both automatic capture and top–down guidance in the same paradigm, we employed a procedure used previously to assess attentional guidance (Downing, 2000; Soto et al., 2005). Participants are first presented with a cue, which is either a food or non-food item, followed by a search display. The cue either has to be identified (the bottom–up priming condition) or it has to be held in memory for a later memory test (the top–down working memory condition). The initial cue can either not reappear in the next search display, or reappear next to or in the opposite field to the search item. When this happens, the re-appearing item can capture the participants’ attention, even when it is irrelevant to the subsequent selection task. Interestingly, this re-appearance effect was typically much stronger when the first item was held in working memory than when it was merely identified (in the bottom–up priming condition) and it was stronger for food items than non-food items (Higgs et al., 2012; Rutters et al., 2014). Like the bottom–up priming effect, the effect of working memory stimulus can be involuntary, influencing search even when it does not predict the search target (Soto et al., 2005). In our current study, we used this paradigm to assess how automatic capture and top–down guidance to food items is modulated by individual variation in successful self-control in a group of healthy participants.

## Materials and Methods

### Participants

The Research Ethics Committee of Birmingham University approved the study, which conformed to the Declaration of Helsinki. Written consent was obtained from all participants. We included healthy and medication-free men and women, with no restrictions on age or BMI, who took part in our experiment for course credit or cash. All participants had normal to corrected-to-normal-vision. Sample size was determined *a priori* on the basis of the effect sizes obtained in our previous studies (Higgs et al., 2012; Rutters et al., 2014).

### Task Description

**FIGURE 1:**
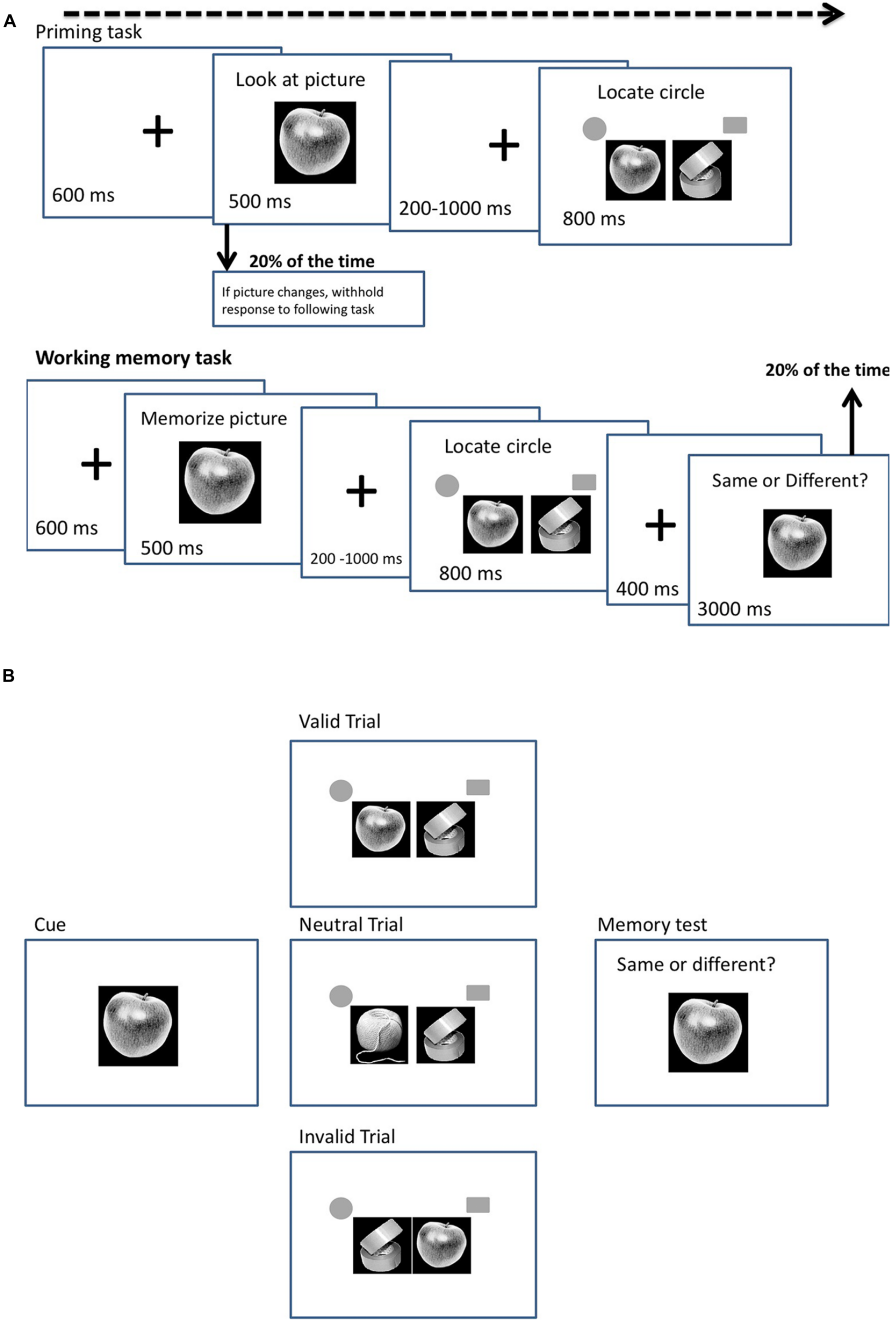
**(A)** Design for priming and working memory tasks. **(B)** Example of working memory task, representing a food valid, food neutral and food invalid trial.

Our experiment consisted of two tasks, a priming task and a working memory task, which varied only in task instructions. In the priming task, participants were asked to identify the cue but not to hold it in memory. In the working memory task, participants were asked to hold the cue in memory across the trial in order for it to be matched in a subsequent memory test. Both tasks contained valid, neutral and invalid trials, a total of 650 trials for each task. On each trial, participants were presented with either a food or a non-food cue (**Figures 1A,B**). The cue was either a picture of a food item, a household item or a stationery item and 10 different pictures per category were used during both tasks. A trial started with a central fixation cross for 600 ms, followed by a cue for 500 ms. After the cue, a fixation cross appeared for 200–1000 ms (randomly chosen), followed by the search array, which consisted of a target (a circle) and a distractor (a square) that appeared randomly to the left or right of fixation (see **Figure 1A** for an example of a trial in the priming and working memory task). Participants had to press ‘c’ if the circle appeared on the left and ‘m’ if it appeared on the right, with the maximum response time set at 800 ms. The target and the distractor were each flanked by a picture of a food item, a household or a stationery object. The inter-trial interval was 400 ms. In the working memory task, 20% of the trials ended with a memory probe that followed the search display to check that the participants were performing the task correctly and had remembered the cue as instructed. On the memory probe trials an item from the same category as the cue appeared for 3000 ms and the participants indicated whether the item was the same or different to the cue. Participants pressed ‘c’ if the item matched the cue or ‘m’ if it was different. No memory probes were presented for the priming task; however, in the priming task the cue disappeared after 250 ms on 20% of the trials and a different image appeared in its place. On these trials, participants were required to withhold their response to the search the task.

On valid trials, the target was flanked by an image that was the same as the cue and the distractor in the search display was flanked by an image from one of the other cue categories. On invalid trials, the distractor was flanked by an image that was the same as the cue and the target was flanked by an image from one of the other cue categories. On neutral trials, both the target and distractor were flanked by images from categories different from the cue (see **Figure 1B** for an example of the working memory task, representing food valid, food neutral, and food invalid trials). The trials occurred randomly with equal probability. All pictures were matched on visual characteristics, presented in black and white, sized 480 × 480 pixels and appeared in the middle of the screen with a black background. A preliminary analysis failed to find any differences in reaction times (RTs) according to whether stationery or household items flanked targets and distractors, on neutral trials (*P* < 0.15). In subsequent analyses the data for these two categories were pooled.

### Apparatus

The tasks were presented using E-Prime (Version 1.2 – Psychology Software Tools) on a SyncMaster 793s color monitor (SAMSUNG, Seoul, Korea). The monitor resolution was 1024 × 768 pixels and the frame rate was fixed at 85 Hz.

### Data Processing

We removed incorrect responses and RTs that were ±3 SDs from the mean. In both the priming and working memory task, the accuracy for the search task was high; an average of 96% correct. In the priming task, responses on catch trials were withheld as instructed with an average of 90% correct; in the working memory task, responses to the memory task were correct in 84% of all cases. There was no evidence of a speed–accuracy trade off.

### Three Factor Eating Questionnaire (TFEQ)

To assess successful dietary self-control, participants filled out the TFEQ that measures three components of eating behavior after completing the priming and working memory tasks (Stunkard and Messick, 1985). The first factor measures dietary restraint that reflects the extent to which individuals attempt to cognitively control their food intake. The second factor measures tendency toward disinhibition of restraint, which reflects loss of control over eating in response to the presence of palatable food or other disinhibiting stimuli, such as emotional distress. The third factor measures the subjective feeling of hunger. We analyzed the effects of restraint and disinhibition by comparing groups based on median splits of the data, participants were characterized as unrestrained when dietary restraint scores were <9 and as having low disinhibition when disinhibition scores were <7 (Westerterp-Plantenga et al., 1991). Participants with high restraint and high disinhibition scores are often referred to as being ‘unsuccessful dieters,’ while those with high restraint and low disinhibition scores are often referred to as being ‘successful dieters’ (Yeomans et al., 2004).

### Procedure

The experiment took place in the morning and participants were asked to refrain from eating before attending (overnight fast). When the participants arrived in the lab, they were asked to describe and write down what they normally have for breakfast and what they had for breakfast today. If the participants ate before attending, they were asked to reschedule. At the start of the experiment, participants rated their feelings of hunger and satiety using a 100 mm Visual Analog Scale. After this, the priming and working memory tasks were completed in a counterbalanced order. Before leaving, participants rated their feelings of hunger and satiety again, and rated the pictures presented in the tasks for liking (‘how much do you like this item in general’), wanting (‘how much do you want this item right now’), and attractiveness (‘how attractive does the item in the picture look’) using Visual Analog Scales. Finally, participants completed the TFEQ and had their height (cm) and weight (kg) measured.

### Analysis

Statistical analyses were performed with SPSS version 20.0 (SPSS Inc., IBM Corporation, Armonk, New York, NY, USA). Continuous data were presented as means ± SEM. To confirm our previous findings on food items modulating ‘top–down’ factors to guide attention, we carried out a 2 × 3 × 2 repeated-measures ANOVA with the factors task (priming, working memory), validity (valid, invalid, neutral) and cue (food, non-food). To assess the differences between items in modulating ‘top–down’ attention to food cues for the dietary self-control groups, we carried out a 2 × 2 × 2 × 2 repeated-measures ANOVA with the factors task (priming, working memory), cue (food, non-food), restraint score (median split < 9) and tendency towards disinhibition score (median split < 7) in valid trials. To correct for possible confounding, we adjusted for BMI and gender. To decompose the interaction between task, validity and cue, we assessed differences in the food advantage scores [%RT for (non-food minus food)/non-food] in valid trials. One-way ANOVAs were used to decompose interactions. Finally, we performed sensitivity analysis, repeating the 2 × 2 × 2 × 2 repeated-measures ANOVA with the factors task, cue, restraint score (median split < 9) and tendency towards disinhibition score (median split < 7) excluding the participants with a restraint score between 8 and 9 as well as participants with a disinhibition score between 6 and 7. Additionally, we carried out the 2 × 2 × 2 × 2 repeated-measures ANOVA with the factors task, cue, restraint score (median split < 8) and tendency towards disinhibition score (median split < 6).

## Results

### Participant Characteristics

All 69 participants were included in the analyses and had a mean age of 21 years, range 18–33 years and mean BMI of 24.1 kg/m^2^, range 15–37 kg/m^2^. Mean hunger and fullness scores at the start of the experiment were 60.7 ± 25 mm and 19.1 ± 19 mm, which suggests that participants were moderately hungry. Average TFEQ scores for restraint, disinhibition and hunger were 8.2 ± 5, 6.7 ± 3, and 5.9 ± 3, respectively.

The participant’s characteristics grouped by restraint score (median split < 9) and tendency toward disinhibition score (median split < 7) are described in **Table 1**. We observed no differences between the groups, except for the high restraint, high disinhibition group (the unsuccessful dieters) being more often female and having a higher BMI, compared to the control participants.

**Table 1:** Participant’s characteristics grouped by restraint score (median split < 9) and tendency towards disinhibition score (median split < 7; *n* = 69).

	Low restraint, low disinhibition	Low restraint, high disinhibition	High restraint, low disinhibition	High restraint, high disinhibition	*P*-value
*N*	21	17	13	18	
Age (years)	20.5 ± 1	19.7 ± 1	22.0 ± 1	21.6 ± 1	0.20
Sex (% male)	62^∗^	53	54	28	0.20
BMI (kg/m^2^)	22.5 ± 1^∗^	22.4 ± 1^∗^	25.6 ± 1	26.5 ± 1	0.01
Hunger (mm)	64 ± 4	58 ± 7	65 ± 7	56 ± 7	0.65


*Mean ± SEM. ^∗^*P* < 0.05 Significantly different from high restraint, high disinhibition group*.

### Task Performance

**Table 2** presents the mean reaction times (RTs) in milliseconds to food and non-food cues, for valid, invalid and neutral trials in the priming task and working task. We first carried out a 2 × 3 × 2 repeated-measures ANOVA with the factors task (priming, working memory), validity (valid, invalid, neutral) and cue (food, non-food). RTs were longer in the WM than the prime task [*F*(1,68) = 91.9; *p* < 0.001, $\eta_{p}^{2}$ = 0.6], suggesting that the participants were performing the priming and WM task differently. There was also a main effect of validity [*F*(2,136) = 145.8; *p* < 0.001, $\eta_{p}^{2}$ = 0.7], whereby RTs were shorter for valid trials compared to neutral and invalid trials and they were shorter for neutral compared to invalid trials (all *p* < 0.05). There was also a main effect of cue [*F*(1,68) = 40.2; *p* < 0.001, $\eta_{p}^{2}$ = 0.4], whereby RTs for food cues were shorter compared to RTs for non-food cues. However, there was a two-way interaction between task and cue [*F*(1,68) = 7.1; *p* < 0.01, $\eta_{p}^{2}$ = 0.1]; RTs were shorter for food cues in both the priming and WM task, however the difference was smaller in the priming task (*P* < 0.01). In addition, there was a significant two-way interaction between task and validity [*F*(2,136) = 37.5; *p* < 0.001, $\eta_{p}^{2}$ = 0.4]; RTs were shorter for valid trials compared to invalid trials (*p* < 0.001), as well as the neutral trials (*p* < 0.001) in the WM task. We observed a similar pattern in the priming task, however, the effect was smaller and only the difference between valid and neutral trials was reliable (*p* < 0.05). Finally, the two-way interaction between validity and cue was also significant [*F*(2,136) = 25.9; *p* < 0.001, $\eta_{p}^{2}$ = 0.3]; RTs were shorter following food cues compared to non-food cues in the valid trials, while no differences were observed in the neutral and invalid trials (*p* < 0.001). The three-way interaction between task, validity, and cue [*F*(2,136) = 0.58; *p* = 0.56, $\eta_{p}^{2}$ = 0.009] was not significant.

**Table 2:** Mean reaction times (milliseconds) and 95% confidence intervals to food and non-food cues, for valid, invalid and neutral trials in the priming task and working memory task (*n* = 69).

	Priming task			Working memory task		
	Valid trials	Neutral trials	Invalid trials	Valid trials	Neutral trials	Invalid trials
Food cue	474.3 (460–488)	495.8 (480–511)	509.7 (493–527)	503.7 (490–517)	537.6 (522–553)	558.9 (543–575)
Non-food cue	487.1 (473–502)	497.7 (482–514)	506.2 (489–523)	520.1 (506–534)	537.6 (530–562)	558.5 (542–575)


The overall data indicate that cues held in working memory had a greater effect on subsequent stimulus selection than cues that were merely identified – this matches prior research (e.g., Soto et al., 2005). Irrespective of whether they were held in working memory or not, food cues exerted stronger effects than non-food cues on subsequent selection also in line with previous findings (Higgs et al., 2012; Rutters et al., 2014). In addition, the effect of working memory load (the longer all-round RTs when cues were held in working memory compared with when they were merely identified) was reduced for food compared with non-food items. The three-way interaction was likely masked by variations in successful self-control, which we examine in detail next.

### Effects of Dietary Self-Control

To assess whether there are differences in working memory modulation of attention to food cues between the four groups of dietary self-control, we compared RTs for food and non-food cues in the priming and working memory task for the valid trials only. This was because there were no previous effects of food cueing for the invalid and neutral trials. **Table 3** presents the mean RTs in milliseconds to food and non-food cues, for valid trials in the priming task and working memory task, stratified for those with restraint score (median split < 9) and tendency towards disinhibition score (median split < 7). We carried out a 2 × 2 × 2 × 2 repeated-measures ANOVA with the factors task (priming, working memory), cue (food, non-food), restraint score (median split < 9), and tendency towards disinhibition score (median split < 7). We observed that RTs were longer in the working memory task than the priming task [*F*(1,65) = 48.9; *p* = 0.001, $\eta_{p}^{2}$ = 0.43] and that RTs for food cues were shorter compared to non-food cues [*F*(1,65) = 61.5; *p* = 0.001, $\eta_{p}^{2}$ = 0.49). We did not observe any interaction effects between task, restraint scores and disinhibition scores nor between cue and restraint scores. We did, however, observe an interaction between cue and disinhibition [*F*(1,65) = 8.7; *p* = 0.004, $\eta_{p}^{2}$ = 0.12). This suggests that participants with high disinhibition scores have shorter RTs for food cues than non-food cues, relative to those with low disinhibition scores, regardless of task. We did not observe any significant interactions between task and cue nor any three-way interactions between task cue and restraint or disinhibition scores, but there was a four-way interaction between task, cue, restraint, and disinhibition score [*F*(1,65) = 4.1; *p* = 0.04, $\eta_{p}^{2}$ = 0.06]. BMI is not a significant confounder of this interaction, while the effect was still present after correction [*F*(1,64) = 3.9; *p* = 0.05, $\eta_{p}^{2}$ = 0.06]. Similarly, the correction for gender did not change the four-way interaction [*F*(1,64) = 4.3; *p* = 0.04 $\eta_{p}^{2}$ = 0.06].

**Table 3:** Mean reaction times (milliseconds) and 95% confidence intervals to food and non-food cues, for valid trials in the priming task and working memory task (*n* = 69), stratified for those with restraint score (median split < 9) and tendency toward disinhibition score (median split < 7).

	Priming task		Working memory task	
	Food cue	Non-food cue	Food cue	Non-food cue
Low restraint, low disinhibition (*n* = 21)	466.1 (441–491)	473.2 (447–499)	484.8 (458–511)	501.5 (475–528)
Low restraint, high disinhibition (*n* = 17)	483.7 (451–516)	500.3 (466–536)	520.2 (492–549)	537.9 (505–571)
High restraint, low disinhibition (*n* = 13)	485.2 (440–494)	493.4 (458–515)	521.2 (495–546)	524.4 (491–552)
High restraint, high disinhibition (*n* = 18)	466.9 (454–516)	486.5 (456–530)	497.6 (469–525)	521.4 (496–552)

**FIGURE 2:**
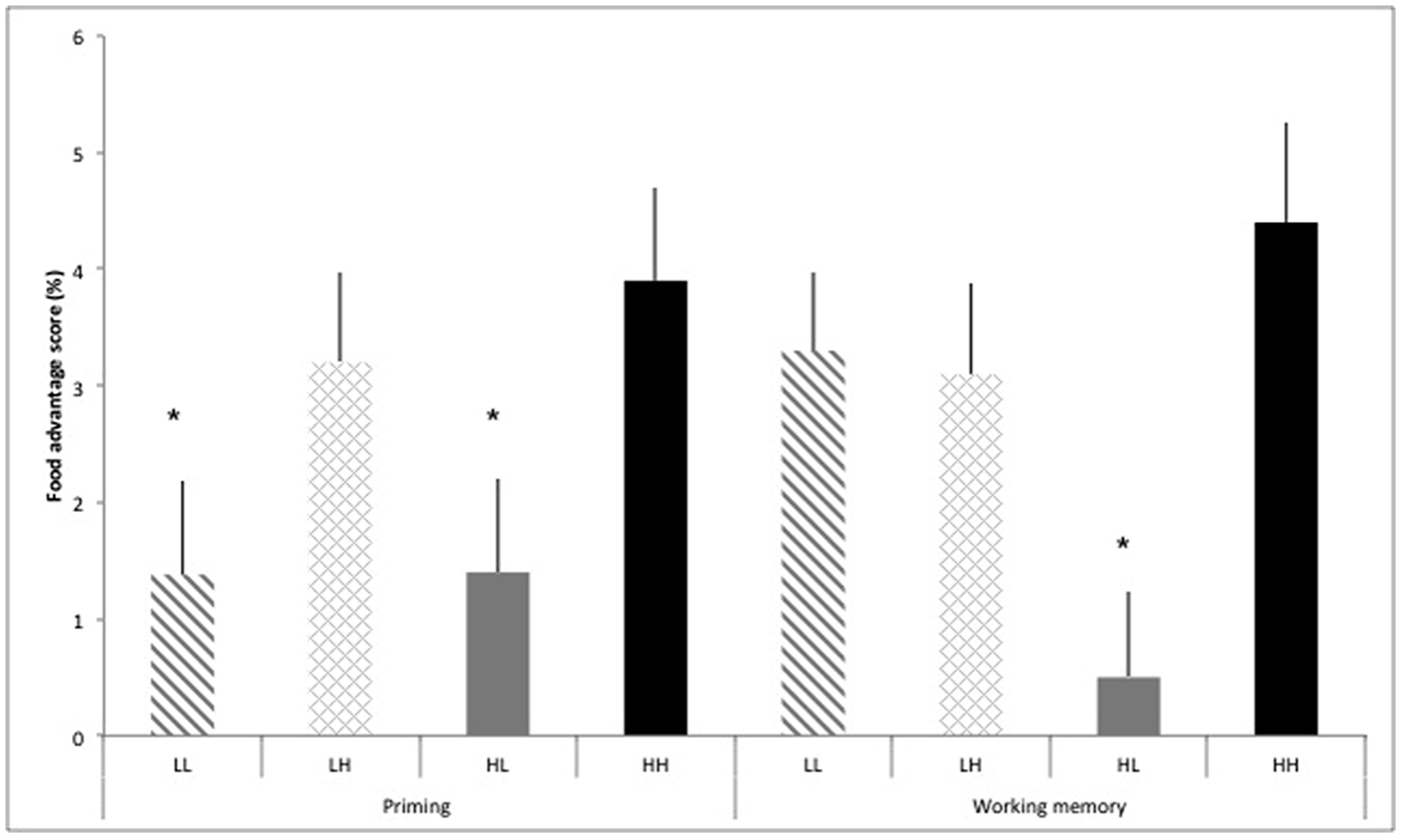
Food advantage [calculated as reaction times for food minus non-food cues/food*100] (%) for valid trials in the priming task and working memory task stratified by groups of dietary self-control: low restraint-low disinhibition scores (LL), low restraint-high disinhibition scores (LH), high restraint-low disinhibition score (HL), and high restraint-high disinhibition scores (HH). Values are means ± SEM. *P < 0.05 food advantage.

To decompose this four-way interaction, we assessed differences in the food advantage scores [%RT for (non-food minus food)/Non-food] for the priming task and working memory task stratified by the four groups of dietary self-control (**Figure 2**). The food advantage score reflects the additional attention paid to food versus non-food after exposure to food cues. For the priming task, there was no significant overall effect for differences between the groups of dietary self-control (*p* = 0.06). However, the food advantage effect was significant in unsuccessful dieters (HH; high restraint and high disinhibition scores, *p* = 0.001) and participants scoring low in restraint and high in disinhibition (LH, *p* = 0.002). There was no significant food advantage effect in participants scoring low in both restraint and tendency towards disinhibition (*p* = 0.15) as well as successful dieters (HL; high restraint and low disinhibition scores, *p* = 0.19). For the working memory task, there was a significant overall effect for differences between the groups of dietary self-control (*p* = 0.01). The food advantage effect was significant in participants scoring low in both restraint and tendency toward disinhibition (LL, *p* = 0.001), those scoring low in restraint and high in disinhibition (LH, *p* = 0.001) as well as unsuccessful dieters (HH; high restraint and high disinhibition scores; *p* = 0.001), while there was no food advantage effect in successful dieters (HL; high restraint and low disinhibition scores, *p* = 0.60).

### Sensitivity Analysis

To stratify the participants in the four groups of dietary self-control, we used the frequently used but arbitrary median cut-off point in restraint scores and disinhibition scores (Westerterp-Plantenga et al., 1991; Yeomans et al., 2004). We therefore performed several sensitivity analyses. First, we carried out a 2 × 2 × 2 × 2 repeated-measures ANOVA with the factors task, cue, restraint score (median split < 9), and tendency towards disinhibition score (median split < 7), excluding the participants with a restraint score between 8 and 9 as well as participants with a disinhibition score between 6 and 7. We observed similar associations and interactions compared to the original analysis, with the four-way interaction between task, cue, restraint, and disinhibition score still being significant [*F*(1,40) = 4.04; *p* < 0.05, $\eta_{p}^{2}$ = 0.09]. Secondly, we carried out a 2 × 2 × 2 × 2 repeated-measures ANOVA with the factors task, cue, restraint score (median split < 8), and tendency towards disinhibition score (median split < 6). We observed similar association and interaction compared to the original analysis, with the four-way interaction between task, cue, restraint and disinhibition score being borderline significant [*F*(1,65) = 3.48; *p* < 0.056, $\eta_{p}^{2}$ = 0.05].

## Discussion

In agreement with previous work, we observed that holding a cue in working memory before a search task modulated the allocation of visual attention (Downing, 2000; Soto et al., 2005; Higgs et al., 2012). This is consistent with working memory having a modulatory influence on visual attention, directing attention to items that match the content of the memory representation. Reaction times were also generally longer in the working memory condition, compared with the priming condition. This again matches prior work and is consistent with participants carrying a greater cognitive load under the working memory conditions (Downing, 2000; Soto et al., 2005; Higgs et al., 2012). Prior work indicates that this effect of cognitive load is independent of the effect of the cue on subsequent visual selection (Tsvetanov et al., 2012). There was also a greater difference between food cues and non-food cues in the working memory task compared with the priming task. Apparently the main effect of memory load was reduced for food items; this is consistent with food items being easier to maintain in working memory (Higgs et al., 2012; Rutters et al., 2014). However, the greater effect of working memory task than priming on visual selection was not greater for food than for non-food cues (there was no task × validity × cue interaction). The reason for this was clarified by the subsequent analyses taking into consideration individual differences in dietary self-control.

Dietary self-control was measured using the TFEQ (Stunkard and Messick, 1985). We observed different patterns of responding according to these factors. For participants scoring high in tendency towards disinhibition in eating (low in self-control), exposure to food cues enhanced attention to food-related stimuli, but this held irrespective of whether the food item were merely identified (in the priming condition), or held in working memory. This suggests that, for these participants, food representations may always be in a potentiated state, easily primed just by the sight of food. Strikingly, successful dieters demonstrated a weak attentional advantage for food in the working memory task. Prior work on the relations between working memory and attention have shown that items in working memory can be suppressed under certain conditions – notably when the item only ever predicts a distractor and participants have sufficiently long to suppress the working memory representation (Han and Kim, 2009). Here it appears that successful dieters tend to inhibit the representation of food in working memory. This reduces the impact of food items that subsequently re-appear in a search display. We conclude that successful dieting is linked to inhibition of food in working memory. It is possible that exposure to food-related thoughts elicits counteractive motivational mechanisms in successful dietary restraint (Fishbach et al., 2003). It has been suggested that successful dietary restraint may depend upon the ability to respond to food cues by activating long-term dietary goals (Papies et al., 2008; Ouwehand and Papies, 2010). It is tempting then to suggest that successful dieters are able to inhibit food items in working memory and this mechanism may be a marker for successful restraint. These results are significant because they may have implications for the design of interventions to help people who find restraint difficult.

In summary, we confirm that the processing of food-related information in working memory can be particularly effective in guiding attentional selection of food stimuli, but this result depends on individual differences in dietary self-control. We find that there are individual differences in attentional guidance such that high levels of disinhibition are associated with generally faster responses to food stimuli, but in the absence of this tendency, dietary restraint protects against the effects on attentional selection of holding food-related information in working memory. An important question for future research is to assess whether these individual differences in attentional guidance to food may determine the effectiveness of dietary interventions.

## Conflict of Interest Statement

The authors declare that the research was conducted in the absence of any commercial or financial relationships that could be construed as a potential conflict of interest.
